# Understanding Negative Results in tDCS Research: The Importance of Neural Targeting and Cortical Engagement

**DOI:** 10.3389/fnins.2017.00707

**Published:** 2017-12-12

**Authors:** Aurore Thibaut, Ross Zafonte, Leslie R. Morse, Felipe Fregni

**Affiliations:** ^1^Department of Physical Medicine and Rehabilitation, Neuromodulation Center, Spaulding Rehabilitation Hospital, Harvard Medical School, Harvard University, Boston, MA, United States; ^2^Coma Science Group, GIGA-Consciousness, University Hospital of Liege, University of Liege, Liege, Belgium; ^3^Spaulding-Harvard SCI Model System Center, Spaulding Rehabilitation Hospital, Boston, MA, United States; ^4^Massachusetts General Hospital, Harvard Medical School, Harvard University, Boston, MA, United States; ^5^Brigham and Women's Hospital, Harvard Medical School, Harvard University, Boston, MA, United States; ^6^Rocky Mountain Regional Spinal Injury System, Craig Rehabilitation Hospital, Englewood, CO, United States; ^7^Department of PMR, University of Colorado School of Medicine, University of Colorado, Aurora, CO, United States

**Keywords:** tDCS, neuromodulation, clinical trial design and conduct, neural engagement, brain networks

In a recent clinical trial we demonstrated the analgesic effects of anodal transcranial direct current stimulation (tDCS) in patients with spinal cord injury (SCI); however, the positive impact of tDCS on pain was not paralleled by an improvement in quality of life or other related clinical scales. Here we discuss the reasons of such *negative* results and present hypotheses that could explain why tDCS had no impact on patients' quality of life, while their average level of pain decreased. We will also discuss how these *negative* findings can help to design future clinical trial using tDCS to treat individuals with chronic pain.

tDCS is an alternative but relevant therapeutic option to manage pain and stimulate motor recovery in patients with SCI. This non-invasive neuromodulation technique has been shown to significantly and sustainably reduce pain if applied repeatedly in various pain syndromes (Fregni et al., 2006a,b; Valle et al., 2009; Sakrajai et al., 2014; Castillo-Saavedra et al., 2016). In our previous study we assessed the effects of motor (M1) anodal tDCS on pain relief, as well as satisfaction with life as measured by the Satisfaction with Life Scale (SWLS) (Diener et al., 1985; Dijkers, 1999), quality of life through mental state (Kroenke et al., 2001) with the Patient Health Questionnaire (PHQ-9), and symptoms of depression with the Beck Depression Inventory. The study comprised two phases; the first one consisted of five tDCS sessions with behavioral assessments completed at baseline, at the end of the five stimulation sessions, at 1-week and 3-month follow-up. The second phase started after the end of the 3-month follow-up period of the Phase I. During this second phase, patients who agreed to continue received 10 sessions of tDCS (applied once daily for 2 weeks) in order to evaluate the effects of adding a second phase of treatment. Assessments were performed after 5 and 10 stimulation sessions and at 2, 4, and 8-week follow-up. While pain was found to be reduced in the active treatment group, and this effect was maintained up to 4 weeks after the last tDCS session, quality of life and mood remained unchanged throughout the entire duration of the protocol for both active and sham groups.

Several hypotheses could explain why anodal M1 tDCS had no influence on mood or satisfaction with life: (1) M1 may not be an appropriate target to modulate mood or satisfaction with life; (2) the primary outcome was pain and therefore this study was not designed to specifically detect change in such scales; (3) the SWLS and PHQ-9 may not be accurate or sensitive enough to detect changes due to tDCS treatment.

Based on the current tDCS literature and the present understanding of pain sensitization mechanisms, the sensorimotor cortex is the most relevant area to target when aiming at treating pain (Castillo Saavedra et al., 2014). By stimulating this specific brain region, anodal tDCS can reduce or reverse the detrimental reorganization of the neural pain network occurring as a consequence of chronic pain mechanisms (Seifert and Maihöfner, 2009; Henderson et al., 2011; Gustin et al., 2012). Several studies have shown that M1 stimulation, using either tDCS or other non-invasive brain stimulation techniques such as transcranial magnetic stimulation (TMS), leads to local and distant neural effects that result in pain reduction (Lefaucheur, 2016). For instance, stimulating M1 may counteract the lack of inhibition from M1, that could also be associated with pain reduction (Botelho et al., 2016; Caumo et al., 2016). In addition, neuroimaging studies have demonstrated that tDCS could also induce changes in thalamic, insula and cingulate activity (Yoon et al., 2014; Simis et al., 2015), known to be related to pain processing (Treede et al., 1999). Regarding quality of life and life satisfaction, significant improvements have been observed when tDCS was applied over the dorsolateral prefrontal cortex (DLPFC), rather than M1 stimulation, in various conditions (Fregni et al., 2006b; Mori et al., 2011; Viana et al., 2014; Liu et al., 2016). Indeed, the prefrontal region has been shown to be a better modulator for mood, and quality of life (Schaffer et al., 1983; Drevets, 1998). The region of interest, and subsequently the network to target, is one of the most important parameters when designing a clinical trial, based on the symptoms or behaviors to improve. In this context it is essential to evaluate the brain behavior relationship with the appropriate tools when designing a clinical trial. This can be seen as a decisional pyramid between: symptom(s)—network to target—behavioral correlates. In this context it is critical to have a clear *a priori* hypothesis and to select the appropriate clinical scale(s) accordingly. A recent framework developed by researchers from National Institute of Mental Health (NIMH), called the research domain criteria (RDoC) is useful for such analysis.

Sankarasubramanian et al. (2017) assessed the effects of anodal tDCS applied on two distinct sites on functional connectivity. They found site-specific effects of M1 tDCS vs. tDCS applied over the DLPFC on functional connectivity between the thalamus and the cortex. Both M1 and DLPFC tDCS increased functional connectivity between the ventroposterolateral nucleus of the thalamus and the sensorimotor cortices; however, the connectivity was greater with M1 tDCS. On the other hand, both M1 and DLPFC tDCS increased functional connectivity between the medial dorsal nucleus of the thalamus and the motor cortex, but only DLPFC tDCS modulated functional connectivity between this nucleus and affective cortices (Sankarasubramanian et al., 2017); highlighting the network-specific effect of site-specific tDCS. From a clinical perspective, similar observations have been made. Roizenblatt et al. (2007) compared the effects of anodal tDCS applied over the left DLPFC with M1 tDCS on pain level and sleep in patients with fibromyalgia (Roizenblatt et al., 2007). M1 stimulation was the only condition associated with reduced pain, and it improved sleep quality as well. On the other hand, DLPFC tDCS did not modulate pain level and it worsened sleep efficiency, stressing the site-specificity of tDCS clinical effects. In addition Boggio et al. (2008) showed that in healthy subjects only M1 stimulation leads to a change of somatosensory perception (Boggio et al., 2008). Indeed, it appears than stimulating M1 mainly influences lateral thalamic projections that process sensory-discriminative information and are paralleled with significant clinical improvement (Antal et al., 2010; Mori et al., 2010), while the stimulation of other sensorimotor structures, such as the somatosensory cortex or the premotor, and supplementary motor areas, have failed to induce similar analgesic effects (Koyama et al., 1993; Hirayama et al., 2006).

Recently, the authors of a randomized controlled trial, notably arguing that they had performed the first and only sufficiently powered trial testing the analgesic effects of tDCS in participants with SCI, concluded that anodal M1 tDCS was ineffective to alleviate or reduce pain in this specific condition when applied for 5 days (Luedtke et al., 2015). In this trial, they compared a group of patients receiving active tDCS for 5 days followed by cognitive training for 4 weeks, to a group of patients who received sham tDCS for 5 days followed by the same cognitive training for 4 weeks. However, such results need to be assessed with caution given that the main area of stimulation (i.e., M1) and the behavioral training that was performed following tDCS (i.e., cognitive behavioral therapy) targeted two different networks. The authors found a significant decrease in pain scores in both groups (sham and active M1 tDCS) after cognitive behavioral therapy, but there were no differences between active and sham tDCS. This result is not surprising since the cognitive behavioral therapy activates mainly prefrontal neural circuits, while M1 tDCS stimulates the sensori-motor network. In this case, an important concept needs to be considered when designing a protocol; it is not only the anatomical region of stimulation that needs to be determined but the anatomical region plus the cortical engagement. For instance, if M1 is targeted with tDCS but a behavioral stimulation activates prefrontal circuits, it is expected that M1 tDCS would have minor, or no effects at all, on enhancing the effects of behavioral stimulation since they target two different circuits. It is suggested that combined therapies should target the same circuit to be additive. Indeed, tDCS could prime the brain targeted circuit before a therapy as it has been shown with combined tDCS and robotic upper limb therapy in cerebral palsy (Friel et al., 2017).

As noted above, it is essential to understand the mechanisms and networks involved in a specific pathology in order to appropriately treat it. It is now well-acknowledged that tDCS modulates not only the area stimulated but also the entire neural network. For instance, by means of neuroimaging studies (fMRI and Positron Emission Tomography—PET-scan), anodal M1 tDCS has been shown to activate ipsilateral motor areas (e.g., primary, supplementary, or premotor cortices) as well as contralateral or long-distance areas (e.g., frontal cortex, somatosensory regions, posterior parietal cortex) and subcortical areas (anterior cingulate cortex) in healthy controls (Lang et al., 2005; Kwon et al., 2008; Kim et al., 2012). These areas are altered in chronic pain syndromes (Baliki et al., 2011). Yoon et al. (2014) used PET-scan to assess brain metabolism after M1 tDCS in participants with chronic pain. They reported that tDCS induces increased metabolism in the medulla and decreased metabolism in the left DLPFC (Yoon et al., 2014). These various neuroimaging studies demonstrate the effect of M1 tDCS on cortical and sub-cortical pain-related network activity. New multichannel-tDCS devices may be even more effective given that they could target and stimulate different areas involved in the same neural network. Preliminary mechanistic studies have shown the superiority of such approaches as compared to conventional dual tDCS (Fischer et al., 2017). Indeed, it has been demonstrated that, as compared to conventional M1 tDCS montage, a single session of novel eight-electrode montage targeting M1 and its associated network induced more than twice as much increase in M1 excitability, measured by resting state fMRI (Fischer et al., 2017). This result shows the possible superiority of such novel network-based multi-channel tDCS approaches; however, behavioral effects still need to be demonstrated.

When discussing multi-channel tDCS protocols, it is essential to stress the importance of the polarity and electrode montage, together with the optimal current intensity. For instance, in an experiment aiming at studying the effect of increasing current intensities on cortical excitability, Batsikadze et al. (2013), showed that, while cathodale tDCS applied at 1 mA reduced cortical excitability, increasing the intensity to 2 mA induced opposite effects (i.e., enhancement of cortical excitability). These results suggest that increasing the intensity of stimulation does not necessarily strengthen the effects but, on the contrary, can result in a shift of the direction of expected cortical changes. To help clinicians and researchers to test and validate optimal electrodes montage and current parameters before running an experiment, computer-based model of the electrical field should be explored, such as demonstrated recently in several studies (Kessler et al., 2013; Gillick et al., 2014; Galletta et al., 2015).

Regarding the selection of the main outcome measure(s), especially when it concerns clinical scales, it is of an upmost importance to choose a sensitive tool that accurately evaluates the symptoms being treated and/or the mechanisms of the therapy. In our case, the mental states evaluated by the selected scales (i.e., QoL and SWLS) are unrelated to modulation of the motor cortex excitability. The expected improvements would have been related to the indirect effects of pain relief on patients' quality of life and satisfaction. Despite the fact that many studies have demonstrated a correlation between pain levels and quality of life (Wahl et al., 2009; Scholich et al., 2012; Wranker et al., 2014), it cannot be assumed that a novel treatment for pain will also effectively treat associated secondary manifestations. This brings us to another factor critical to be considered when analyzing the results of a trial. The fact that no improvements were noticed on secondary outcomes does not conclusively demonstrate that the intervention is ineffective for such symptoms. Indeed, a trial is designed based on the primary outcome measure. The protocol design, sample size calculation, follow-up measures, or the chronology of the tests are all factors that are elaborated based on the research question and the main goal of a study. For instance, it is possible that our study was not sufficiently powered to detect changes in QoL or life satisfaction. Other scales, such as the SF-36 (36-Item Short Form Survey, assessing mental state based on patients' report; McHorney et al., 1993) may have been more sensitive to detect changes since they may be more impacted by pain. Similarly, the effects we found in pain scores in our trial may not have been strong enough to modify other related outcomes. In addition, to observe improvement on mental states, especially in individuals with chronic pain, a greater and more prolonged effect on pain may be required, and therefore, more tDCS sessions may be needed to induce significant quality of life enhancements. It is also important to objectively analyze the (negative) results and explain such findings, while avoiding over-concluding if the pre-specified hypothesis is not met. To conclude, we here aimed to emphasize the importance of network selection when designing a clinical trial on tDCS effects. This requires a good understanding of the mechanisms underlying the symptoms to treat and the neural adaptation occurring in the related condition. For chronic pain, evidence suggests that pain is associated with the phenomenon of central sensitization involving a large neural network that includes limbic structures, such as the anterior cingulate cortex, hippocampus and amygdala, and thalamic nuclei, as well as the sensorimotor area (Treede et al., 1999; Phillips and Clauw, 2011; Bourke et al., 2015). In addition, enhanced activity in pain-related brain structures have been reported, including the motor cortex (Fregni et al., 2006a). By targeting an area that is linked to pain circuitry, we expected to observe significant effects of tDCS on pain level as measured by the visual analog scale (VAS) and we confirm our hypothesis. Similar *symptoms-network-outcome* pyramidal approach needs to be applied when designing a clinical trial on neuromodulation. Moreover, behavioral intervention(s) targeting the same cortical network should be tested in addition to tDCS in order to prime its effects.

## Author contributions

AT and FF: wrote the manuscript. RZ and LM: significantly reviewed the manuscript for intellectual content and approved the final version of the manuscript.

### Conflict of interest statement

The authors declare that the research was conducted in the absence of any commercial or financial relationships that could be construed as a potential conflict of interest.
